# Remarks on Venesection in Typhus, as Recommended by Dr. Armstrong

**Published:** 1822-11

**Authors:** W. Norman


ART. V.—*Remarks on Venesection in Typhus, as recommended by* DR. ARMSTRONG. By W. NORMAN, Esq.

FEW men, if any, merit the reward of universal approbation, or the enjoyment of professional celebrity, more than Dr. Armstrong. He has well earned the distinguished reputation he sustains in society;—his whole career has displayed him the zealous cultivator of medical science; and his labours have conferred infinite benefit, not only on the profession to which he is so unquestionable an acquisition, but on the community of which he is so invaluable a member.

Unlike the pedantic, unintelligible lucubrations of some visionary theorists of provincial fame, Dr. Armstrong's works are, for the most part, replete with incontrovertible doctrines, established on the firm basis of philosophical inquiries, matured by practical observations, and, after calm reflection, promulgated to the world, unfettered with prejudices, through the medium of an enlightened understanding, in clear, dignified, and unsophisticated language.

Desiring, most unfeignedly, to apologize to Dr. Armstrong for this unceremonious introduction of his name, associated as it is with the expression of sentiments which, although sincere and unaffected, are weightless and insignificant when thrown into the opposite scale to his well-known deserts, and to you for trespassing so largely on the limits of your Journal, I proceed immediately to the disclosure of my design; which is to summarily discuss the *virtual* import of an abstract sentence found in one of Dr. Armstrong's best productions,—to wit, his work on Typhus Fever, inasmuch as that sentence has, in many instances, been adopted in an individual acceptation, both as a sanction and an apology for a practice that fatal experience has repeatedly shown to be highly dangerous, when *indiscrimi-*

---

*nately* employed in the treatment of the disease to which it relates.

I am aware that your readers may adduce against the expediency of my project (and, *a priori*, with reason too,) the argument, that the sentence alluded to is taken, as it ought to be, in conjunction with, or as referring to, the manifest qualifications of antecedent passages of the same paragraph, so plain and self-explicable as to admit no doubt whatever of the meaning of the learned author himself; and, therefore, that, if its import has been so perverted or mistaken that culpable practice has been instituted in consequence of it, such a circumstance can only have occurred where ignorance and indifference have precluded the dictates of common sense and rational deduction, to the total extinction of every hope of effecting a conversion to truth by argument or demonstration. Nevertheless, possessing, as I do, the facts hinted at in the preceding paragraph, and claiming also to be a member of a profession whose natural attributes and bounden duties are, by competent scientific acquirement, not only to protect the lives of individuals against the destructive incursions of disease, but to guard the community at large against the as destructive practices of prejudiced pretenders, I feel sanctioned in undertaking and prosecuting the avowed object of this communication; and for that purpose hasten directly to quote the paragraph containing the sentence to which the communication particularly refers.

"Perhaps it may be asked why I have not mentioned venesection as a remedy for the strictly typhus? But I may appeal to every practitioner of experience and candour to support me in the assertion, that it may be safely dispensed with in the majority of cases. When typhus appears from the first in its least complicated form, the early adoption of the plan laid down will not only ward off inflammatory symptoms, but those putrid ones which are apt to arise from them; and thus is calculated to prevent the necessity of blood-letting in the second stage, and the free admission of stimulants in the last. At the same time, whenever, in defiance of the means already recommended, there is an early threatening of some visceral inflammation, the immediate employment of general or local blood-letting, promptly followed up by the application of blisters, will generally be found necessary. It may indeed be regarded as an axiom, that bleeding, if it should not do good, will hardly ever do harm, in the *commencement* of febrile diseases.—(ARMSTRONG *on Typhus Fever*, p. 123.)

Now, the sentence to which I have alluded as having given rise to "highly dangerous practice in the treatment of the disease to which it relates," is the sentence that concludes the foregoing paragraph. Upheld by the latitude of construction which the *literal* expression of that sentence, taken abstractedly, and not in reference to any qualifications that precede it, is supposed to admit of, venesection is by some persons *indiscrimi-*

*nately* adopted and pursued, as the "sine qua non" of their practice in every case of typhus fever that presents itself to their notice, as much so when that practice is *not* indicated by the presence of an inflammatory diathesis as when it *is*, equally regardless or ignorant of the consequences that might result from it.

To analyze every sentence of the quoted paragraph which tends to show that Dr. Armstrong, when he said that, "if it [venesection] should not do good, will hardly ever do harm, in the commencement of febrile diseases," did *not* mean to sanction or recommend *invariable and indiscriminate* blood-letting both at the commencement and during the subsequent progress of typhus fever, were an unnecessary occupation of time,—a prodigal waste of the pages of your Journal; for such an assumption of meaning is in itself irreconcilably at variance with his "appeal to every practitioner of experience and candour to support him in the assertion that it may be safely dispensed with in the majority of cases." Indeed, Dr. Armstrong recommends it where *inflammation is early threatened, in defiance of the other means before recommended;* and to imagine that Dr. Armstrong would compromise his well-earned celebrity by seriously attempting to palm on the medical world the silly and gratuitous notion that venesection, when employed as a remedial agent in typhus fever, possesses a negative quality,—and that, whether inflammatory symptoms or a diametrically opposite state of system were present, it would be all one as to the welfare of the patient, for venesection, if it did no good, *could do no harm,*—was to suspect him of the most consummate folly, the most preposterous absurdity.

Further, if we still keep our eyes on the paragraph in question, we shall clearly perceive that Dr. Armstrong virtually desires to *avoid* venesection altogether in typhus fever, if possible, by suggesting that "the *early* adoption of the plan laid down will not only ward off inflammatory symptoms, but those putrid ones which are apt to arise from them, and thus is calculated to *prevent the necessity of blood-letting.*" Now, were Dr. Armstrong really that advocate for invariable and indiscriminate blood-letting, at the commencement and during the progress of typhus, which the infatuated followers of that system tell us he is, I think he would scarcely be so inconsistent, or so indifferent to the prosperity of that measure, as to broach such doctrines as go at once to destroy its reputation as an *universal* remedy, "branch and root."

As far as my own judgment enables me to form a correct opinion on this interesting subject, and it is compatible with the declared object of my undertaking to express it, I am most ready to admit that I believe blood-letting, generally or topically, employed at the commencement of typhus fever, would be commonly followed by advantage to the patient, though unequivocal symptoms of visceral inflammation were not actually present or immediately threatened. This concession, however, is not to be construed as an approval of the system of invariable and indiscriminate general blood-letting, in contradiction to what has been already advanced on that head; for the impressions of past experience must be entirely obliterated by the results of future observations, before I shall subscribe to measures which, to my certain knowledge, have prematurely consigned many a hapless victim to an untimely grave.

Circumstances, for many years past, have conspired to give me frequent opportunities of investigating the various phenomena constituent of typhus fever,—of contemplating its influence on the human constitution,—of inquiring into the nature and cause of its existence,—and of estimating the different modes of treatment that have at different periods been suggested for its cure; but, as the introduction of these topics here would be at variance with the purport of this communication, I will defer troubling you with the discussion of them to another, not far distant, opportunity.

*Langport; Sept. 9th,* 1822.

